# Cerebellar and basal ganglia inputs define three main nuclei in the mouse ventral motor thalamus

**DOI:** 10.3389/fnana.2023.1242839

**Published:** 2023-08-14

**Authors:** Carmen Alonso-Martínez, Mario Rubio-Teves, Diana Casas-Torremocha, César Porrero, Francisco Clascá

**Affiliations:** Department of Anatomy and Neuroscience, Universidad Autónoma de Madrid, Madrid, Spain

**Keywords:** ventral anterior thalamic nucleus, ventral lateral thalamic nucleus, ventromedial thalamic nucleus, internal globus pallidus, substantia nigra pars reticulata, vesicular glutamate transporters, vesicular GABA transporter, cerebellum

## Abstract

The thalamus is a central link between cortical and subcortical brain motor systems. Axons from the deep nuclei of the cerebellum (DCN), or the output nuclei of the basal ganglia system (substantia nigra reticulata, SNr; and internal pallidum GPi/ENT) monosynaptically innervate the thalamus, prominently some nuclei of the ventral nuclear group. In turn, axons from these ventral nuclei innervate the motor and premotor areas of the cortex, where their input is critical for planning, execution and learning of rapid and precise movements. Mice have in recent years become a widely used model in motor system research. However, information on the distribution of cerebellar and basal ganglia inputs in the rodent thalamus remains poorly defined. Here, we mapped the distribution of inputs from DCN, SNr, and GPi/ENT to the ventral nuclei of the mouse thalamus. Immunolabeling for glutamatergic and GABAergic neurotransmission markers delineated two distinct main territories, characterized each by the presence of large vesicular glutamate transporter type 2 (vGLUT2) puncta or vesicular GABA transporter (vGAT) puncta. Anterograde labeling of axons from DCN revealed that they reach virtually all parts of the ventral nuclei, albeit its axonal varicosities (putative boutons) in the vGAT-rich sector are consistently smaller than those in the vGLUT2-rich sector. In contrast, the SNr axons innervate the whole vGAT-rich sector, but not the vGLUT2-rich sector. The GPi/ENT axons were found to innervate only a small zone of the vGAT-rich sector which is also targeted by the other two input systems. Because inputs fundamentally define thalamic cell functioning, we propose a new delineation of the mouse ventral motor nuclei that is consistent with the distribution of DCN, SNr and GPi/ENT inputs and resembles the general layout of the ventral motor nuclei in primates.

## Introduction

The thalamus is a central link in the multiregional motor networks of the brain. It receives massive direct connections from the basal ganglia (BG) system (internal globus pallidus; GPi, or entopeduncular nucleus; ENT, and substantia nigra pars reticulata; SNr), as well as from the cerebellum (deep cerebellar nuclei; DCN; Sidibé et al., 1997; Kelly and Strick, 2003; Kuramoto et al., 2011). The main target of these pathways in the thalamus is the ventral nuclear group (see review Jones, 2007). In turn, the ventral nuclei innervate motor and premotor areas of the cortex and powerfully support persistent activity during motor planning, execution and learning, triggering a rapid reorganization of the motor cortex state and ramping preparatory activity before goal-directed movements, driving rapid and precise motor behaviors (Gao et al., 2018; Guo et al., 2018; Catanese and Jaeger, 2021; Inagaki et al., 2022). Signal computations by thalamic cells are fundamentally determined by their inputs (Groh et al., 2014; Halassa and Sherman, 2019; Acsády, 2022). Hence, in motor disorders such as Parkinson’s, Tourette, or ataxias, abnormal BG or DCN inputs may critically alter ventral thalamic neuron firing, leading to the emergence of clinical symptoms (see reviews Obeso et al., 2002, 2014).

In primates, and particularly humans, thalamic ventral motor nuclei are massive. Despite past nomenclature controversies (see reviews Jones, 2007; García-Cabezas et al., 2023), there is now wide consensus that three main subdivisions can be distinguished based on the distribution of DCN and BG inputs (Hendry et al., 1979; Ilinsky and Kultas-Ilinsky, 1987; Percheron et al., 1996; Jones, 2007; Barbas et al., 2013). A ventrolateral posterior nucleus is the territory innervated by the DCN axons, a ventromedial nucleus and ventral anterior is the territory innervated by the SNr axons and a ventrolateral anterior innervated by internal pallidal axons (see Jones, 2007; Barbas et al., 2013, for reviews).

Mice have recently become key models in motor system research (see review Ellenbroek and Youn, 2016; Goldberg, 2022; Hoebeek and Boele, 2022). However, the precise distribution of BG and DCN inputs in the rodent motor thalamus is still unclear, making it difficult to establish comparisons with primates. In rodents, the BG and DCN input territories seem to be not clearly segregated (Faull and Carman, 1978; Angaut et al., 1985; Deniau et al., 1992; Kuramoto et al., 2011). Moreover, such territories do not align well with nuclei borders. For example, DCN inputs spread beyond the borders of the ventral lateral (VL) nucleus. Likewise, the SNr inputs reach two nuclei that are traditionally distinguished based on cytoarchitectonic criteria, the ventral anterior (VA) and the ventromedial (VM; Deniau et al., 1992; Sakai et al., 1998; Bodor et al., 2008; Kuramoto et al., 2011; Foster et al., 2021). Data about GPi/ENT inputs to the rodent ventral thalamus are fragmentary (Takada et al., 1994; Kha et al., 2000) and they have never been mapped in the mouse.

Here, we mapped the distribution of inputs from cerebellum, SNr and GPi/ENT to the ventral nuclei of the mouse thalamus. To this end, we immunolabeled the neuropil of these nuclei for glutamatergic and GABAergic neurotransmission markers. In addition, we mapped the thalamic territories targeted by axons anterogradely labeled by viral vector injections or tracer in the deep cerebellar nuclei, SNr or GPi/ENT. Besides, we measured and compared axon terminals size in the various nuclei.

## Materials and methods

### Animals

Experiments were performed on adult (60–120 days old, 25–35 g body weight) wild-type C57BL/6 male mice. Animals were bred in our university Animal Facilities. All procedures involving animals were conducted under protocols approved by the university Ethics Committee and the competent Regional Government agency (PROEX175/16 and PROEX179.3/21), in accordance with the European Community Council Directive 2010/63/UE. Animals were housed under standard colony conditions with food and water *ad libitum* under a 12/12 h light/dark cycle. Efforts were made to minimize the number of animals required. In total, fourteen mice were used for anterograde biotinylated dextran amines (BDA) axon labeling, and thirteen further mice were used for immunolabeling experiments.

### Anesthetic procedures

In the animals used for the tracing experiments, anesthesia was induced with an intraperitoneal injection of ketamine (0.075 mg/g body weight) + xylazine (0.02 mg/g body weight), and subsequently maintained throughout the surgical procedure with isoflurane (0.5–1%) in oxygen. Ibuprofen (120 mg/l) was added to the drinking water to ensure analgesia during the postoperative period. At the time of sacrifice, animals were overdosed with sodium pentobarbital (0.09 mg/g body weight, i.p.).

### Anterograde tracing experiments

To anterogradely trace the projections from the deep cerebellar nuclei, zona incerta (ZI) and anterior pretectal nucleus (APT). Animals were placed in a rodent stereotaxic frame (Kopf Instruments, Tujunga, CA, USA), the sagittal midline of the scalp was sectioned and retracted, and a small craniotomy was drilled. Lysine-fixable 10 kDa biotinylated dextran amine (Invitrogen; 3% w/v solution in 0.01 M PB, pH 7.4) was iontophoretically injected in the deep cerebellar nuclei (AP −6.11, ML ± 1.5 DV −2.00; *n* = 10 cases); zona incerta (AP −2.1, ML ± 1.5, DV −4.00; *n* = 2 cases) and anterior pretectal nucleus (AP −2.8, ML ± 1.1, DV −2.6; *n* = 2 cases); 8–20 μm tip diameter, 0.5–0.7 μA current intensity, 40 min injection, cycle 1 s ON/1 s OFF. Using a Dual Current 260 source (World Precision Instruments, Sarasota, FL, USA) a positive current (0.7 μA) was applied. Bregma was targeted and the stereotaxic coordinates were followed based on the Paxinos and Franklin (2019) mouse brain atlas. Following the injection, the micropipette was left in place for 10 min before removal and wound closure. Animals were then allowed to recover from anesthesia and returned to their cages. Animals were euthanized after 7 days.

### Tissue fixation and histologic procedures

All animals were perfused transcardially with 30 ml of saline, followed by 100 ml of 4% paraformaldehyde (diluted in 0.1 M PB, pH 7.4). Brains were removed from the skull and postfixed overnight at 4°C in the same solution. Subsequently, brains were cryoprotected by embedding in 30% sucrose in 0.1 M PB, at 4°C, for 48 h. In the BDA axonal labeling experiments, brains were freeze-sectioned in the coronal plane at 50 μm, and sections were collected in two parallel series. In the first series, after peroxidase activity blocking by incubation in H_2_O_2_ 0.66% (w/v) in 0.1 M PB for 15 min, sections were incubated for 2 h in avidin-biotin-peroxidase (1:100; Vectastain Elite, Vector Laboraries, Newark, CA, USA) diluted in 0.1 M PB. After washing, peroxidase was visualized using the glucose oxidase-3-3′diaminobenzidine (Sigma-Aldrich Química, Madrid, Spain) nickel sulfate-enhanced method (Shu et al., 1988). For labeling localization, the same sections were then counterstained using cytochrome C-oxidase (CyO) histochemistry (Wong-Riley et al., 1978) and finally mounted and coverslipped with DePeX (Serva Electrophoresis GMbH, Heidelberg, Germany). A second series was kept in antifreeze solution at −20°C as a backup.

### Immunolabeling

In the animals used for immunolabeling experiments, sacrifice and perfusion were carried out as described above. Brains were sectioned in either the coronal (*n* = 6), sagittal (*n* = 4) or horizontal (*n* = 4) planes. Sections were single- or double-immunostained using antibodies against different marker molecules. All the antibodies used are commercially available and their host species, manufacturer, and dilutions are indicated in Table 1. We performed immunolabeling against vesicular glutamate transporters type 1 or type 2 (vGLUT1 or vGLUT2), or the vesicular GABA transporter (vGAT) or against the glutamate decarboxylases 65 and 67 (GAD65/67), or against calbindin type 1 (CALB1) 28kD. These methods were applied independently or combined the tissue sections. Following peroxidase activity blockage as above, sections were serially incubated in: (1) 2% Triton X-100 (Sigma-Aldrich Química, Madrid, Spain) 15% normal goat serum (NGS) 11% bovine serum albumin (BSA) in 0.1 M PBS at room temperature (RT) for 2 h; (2) guinea pig anti-vGLUT1, guinea pig anti-vGLUT2, rabbit anti-vGAT or rabbit anti-GAD65/67 polyclonal antibodies, 2% Triton X-100, 5% NGS and 1% BSA in 0.1 M PBS with at 4°C for 48 h; (3) biotinylated goat anti-guinea pig, 2% Triton X-100, 5% NGS in 0.1 M PBS at RT for 2 h; (4) in ABC Elite (Vector Laboraries, Newark, CA, USA) 1:100 in 0.1 M PBS containing 2% Triton X- 100 at RT for 2 h. Multiple PBS rinses were intercalated between the solutions. Finally, peroxidase activity was visualized using a glucose oxidase-diaminobenzidine protocol (Shu et al., 1988). For double immunofluorescence labeling sections were incubated, free-floating, in a solution combining pig anti-vGLUT2 with rabbit anti-vGAT, or with rabbit anti-GAD65/67 (72 h, 4°C). Following multiple rinses, sections were then incubated in the corresponding secondary AlexaFluor-conjugated antibody (Table 1) in 0.1 M PB containing 2% Triton X-100 and 5% NGS, 2 h, RT. After rinsing in 0.1 M PB, sections were mounted onto gelatin-coated glass slides, air dried, dehydrated graded ethanol, defatted in xylene, and coverslipped with DePeX. vGLUT2 puncta sizes were measured from live images at 100× using a Nikon DMX1200 camera attached to the microscope and the NIS-Elements software tools (v3.2; Nikon). For each nucleus, 300 randomly selected puncta from *n* = 2 vGLUT2 immunolabeling experiments were measured.

**TABLE 1:** List of primary and secondary antibodies used in this study.

Antibody	Manufacturer	Dilution
Guinea pig anti-vGLUT1 polyclonal antibody serum	Chemicon, Merck Millipore, catalog #AB5905	1:5,000
Guinea pig anti-vGLUT2 polyclonal antibody serum	Chemicon, Merck Millipore, catalog #AB5905J	1:2,000
Monoclonal mouse anti-CB polyclonal antibody serum	Sigma-Aldrich, catalog #C9848	1:100
Rabbit anti-GAD65/67 polyclonal antibody serum	Chemicon, Merck Millipore, catalog #ABN904	1:100
Rabbit anti-vGAT polyclonal antibody serum	Synaptic Systems, catalog #131002	1:100
Biotinylated goat anti-guinea pig polyclonal IgG	Vector Laboratories, catalog #BA-7000	1:200
AlexaFluor 488-conjugated goat anti-guinea pig polyclonal IgG	ThermoFisher, catalog #A-11073	1:200
AlexaFluor 568-conjugated goat anti-rabbit polyclonal IgG	ThermoFisher, catalog #A-11011	1:200
AlexaFluor 568-conjugated goat anti-mouse polyclonal IgG	ThermoFisher, catalog #A-11004	1:200

### Anterograde BDA labeling analysis

Since axonal varicosities have been shown to contain presynaptic organelles and their overall size correlates with the number and/or strength of synapses they establish (Rovó et al., 2012; Groh et al., 2014; Rodriguez-Moreno et al., 2020; Casas-Torremocha et al., 2022), we measured and compared labeled axon varicosity sizes. To this end, labeled varicose axons were examined and imaged under brightfield optics using 10–40× objectives. Nuclear boundaries were delineated following references provided by the CyO counterstaining. Varicosities were identified as such when their diameter was at least twice that of the adjacent axonal segments. Bouton size was estimated by measuring, for each varicosity focused on the *z*-axis at 100× in a Neurolucida platform (MBF Microsystems, Williston, VT, USA) mounted on a Nikon Eclipse 80i, the cross-sectional (maximal projection) area. The freehand contour mapping tracing and contour measurements tools (Version 2020.1.1 64-bit, MBF Microsystems) were used. Varicosities with cross-section area below the microscope resolution limit (< 0.2 μm^2^) were not included in the analysis. Varicosities were measured in four different DCN BDA injection cases; we examined slices at three different coronal levels of the ventral thalamic nuclei. A total of 600 varicosities from all four cases together, 200 in each nucleus, were measured.

### Confocal microscope analysis

Immunofluorescence analyses were carried out using a Spectral Leica TCS SP5 confocal microscope by sequentially applying argon (488 nm) or diode -pumped solid-state (561 nm) laser lines to ensure complete channel separation. Regions of interest were imaged using a 20× objective. For each region of interest, 10 image stacks were obtained moving the sample in the *z*-axis. Both image stacks and maximal projections were analyzed in separate and merged channels.

### Image analysis of the Allen brain connectome experiments

Two-photon tomography image collections from the Allen Mouse Brain Connectivity Database (Allen Institute for Brain Science, 2011) were examined. These experiments involved relatively large stereotaxic injections of associated adenovirus vector (AAV) vectors to drive the expression of fluorescent proteins preferentially directed to axons. Experiments with injections in either the cerebellum (IDs: 1220493315, 127650431, 168664192, 264096952, 265928489, 286608092, 304474221, 552283801; *n* = 8)^1^ internal globus pallidus (GPi; IDs: 305024724, 539498984, 278501857; *n* = 3) or the substantia nigra pars reticulata (SNr; IDs: 158914182, 175263063, 299895444; *n* = 3) were analyzed. The extension of the transfected neuronal population at the injection site as well as its axonal projections were analyzed. On these images, the interactive machine learning for (bio) image analysis software Ilastik (Version 1.4; Berg et al., 2019) was used to segment the axonal varicosities using the pixel classification method. The software was first trained to recognize varicosities in images taken from some of the above experiments. Once we got the classifier trained, it was left to identify varicosities in an unsupervised manner.

### Experimental design and statistical analysis

From the 10 experiments involving unilateral BDA injections in the DCN, we selected for analysis the four cases in which the tracer deposit did not spread at all to other brainstem structures (4 injections). Comparison of varicosity median cross-sectional areas was performed using the Kruskal–Wallis test followed by the Dunn’s *post-hoc* test for multiple comparison. Kolmogorov–Smirnov test (K–S) was used to compare between different structures the size distribution of varicosities. Statistical analysis was computed using GraphPad Prism version 9.4.1 for Mac OS X (GraphPad Software, San Diego, CA, USA).^2^ The threshold level of significance was set at *p < 0.005, ^**^p < 0.01, ^***^p < 0.001, and ^****^p < 0.0001.

## Results

### Immunolabeling for glutamatergic neurotransmission markers

To estimate the global distribution of different types of afferent terminals in the neuropil of the ventral motor nuclei, we first immunostained against specific neurotransmitter transporters. The DCN are known to be glutamatergic (Schwarz and Schmitz, 1997). The vGLUT2 protein is known to be absent from corticothalamic terminals and be mainly produced by diencephalic and lower brain stem neurons, including the DCN (Fujiyama et al., 2001; Herzog et al., 2001; Hioki et al., 2003; Fremeau et al., 2004; Bopp et al., 2017). Conversely, the BG afferents to the motor thalamus, such as the GPi/ENT and the SNr are known to be GABAergic (Di Chiara et al., 1979; Kilpatrick et al., 1980; MacLeod et al., 1980; Penney and Young, 1981; Ilinsky et al., 1997; Bodor et al., 2008).

As a proxy for glutamatergic axon terminals of subcortical origin, we immunostained against vGLUT2. To prevent possible biases created by using only a single sectioning plane (Jones, 2007), we compared thalamus sections made in the coronal, sagittal or horizontal planes.

**FIGURE 1:**
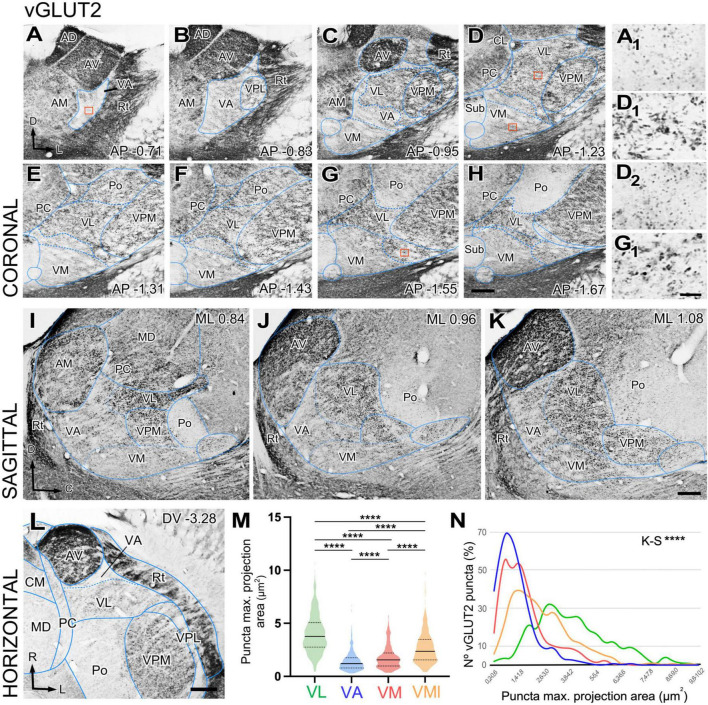
Distribution of the glutamate vesicular transporter type 2 (vGLUT2) immunolabeled puncta in the mouse ventral thalamic motor nuclei. **(A–H)** Coronal section photomicrographs showing in different sections the distribution of terminals marked by immunohistochemistry against the glutamate vesicular transporter type 2 (vGLUT2). Dorsal is to the top and lateral to the right. High-magnification details showing vGLUT2 puncta in VA **(A_1_)**, VL **(D_1_)** medial VM **(D_2_)**, and lateral-caudal VM **(G_1_)**. Note that puncta in VA and the medial portion of VM are smaller and more weakly stained than those in VL or the caudal-lateral portion of VM. Distance from bregma (in mm) is indicated in the lower right corner. **(I–K)** Sagittal photomicrographs showing in different sections the distribution of terminals marked by immunohistochemistry against the glutamate vesicular transporter type 2 (vGLUT2). Dorsal is to the top and caudal is to the right. Distance from the midline (in mm) is indicated in the upper right corner. **(L)** Horizontal section. Note the difference in staining between VA and VL neuropil. **(M,N)** Comparison of immunostained puncta size (maximal projection area in μm^2^) measured in the neuropil of VL, VA, VM and caudolateral portion of ventromedial nucleus (VMl). Violin-plot charts in **(M)** show the density distribution of the data (*N* = 300). Median is indicated by a continuous black line and quartiles by dotted lines. Statistical differences were computed using the Kruskal–Wallis test followed by the Dunn’s *post-hoc* test (****p < 0.001). Panel **(N)** shows the frequency distributions of puncta sizes (Kolmogorov–Smirnov test; ****p < 0.001). Scale bars: 250 μm **(A–L)** and 10 μm (**A_1_,D_1_,D_2_** and **G_1_**).

We observed that the neuropil immunoreactivity for vGLUT2 is markedly heterogeneous. Immunolabeling is heavy in the dorsocaudal portion of the ventral anterior/ventral lateral (VA-VL) complex (henceforth referred to as VL, for simplicity). Likewise, the anterior (AD; anterodorsal, AV; anteroventral), ventral posteromedial (VPM), intralaminar, or reticular prethalamic nuclei (Rt) show heavy vGLUT2 labeling (Figures 1A–L). In contrast, the rostroventral part of the VA-VL complex (henceforth referred to as VA, for simplicity; Figures 1A–C, I–K, L) and the ventromedial nucleus (VM; Figures 1C–K) show weak and scarce immunolabeling. Higher magnification shows that the labeled puncta display different morphologies in the various nuclei. For example, average puncta in VL (Figure 1D1) are significantly larger (4.02 ± 1.8 μm^2^) and more heavily stained than those in VA (1.43 ± 0.9 μm^2^; Kruskal–Wallis and Dunn test: *p* < 0.001; Figure 1A1) or in VM (1.80 ± 1.1 μm^2^; Kruskal–Wallis and Dunn test: *p* < 0.001; Figure 1D2). A small lateral and caudal domain of VM contains larger (2.66 ± 1.5 μm^2^; Kruskal–Wallis and Dunn test: *p* < 0.001) and heavily labeled puncta than the rest of the nucleus (Figures 1G, H, G1). It is of note the vGLUT2 immunolabeling does not reveal a border between VA and medial VM. Distribution of puncta sizes are significantly different between all compared regions (Kolmogorov–Smirnov test: *p* < 0.001) (Figures 1M, N).

As a proxy for glutamatergic axon terminals of cortical origin, we immunostained against vGLUT1. In this case, labeling was uniformly heavy throughout the ventral motor and adjacent nuclei (Supplementary Figures 1A–D). High-magnification analysis revealed no differences between the nuclei in vGLUT1 puncta size or staining intensity (Supplementary Figures 1E–H).

### Immunolabeling for GABA neurotransmission markers

**FIGURE 2:**
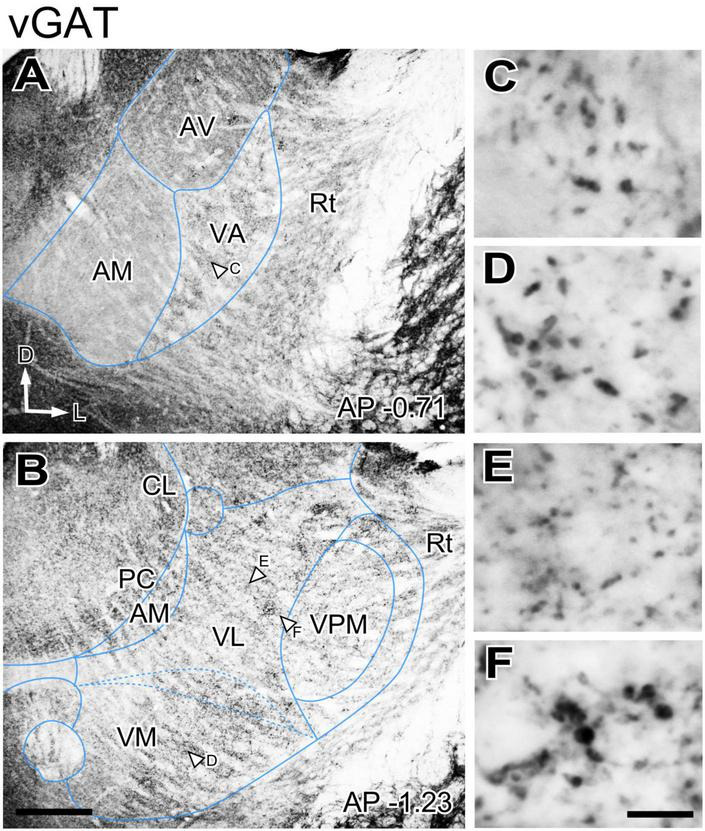
Distribution of the vesicular GABA transporter (vGAT) immunolabeled puncta in the mouse ventral thalamic motor nuclei. **(A,B)** Coronal section images showing the variation in labeled puncta density across the ventral motor nuclei. **(C–F)** High-magnification detail of the labeled puncta. The sampled regions are indicated by white arrows in **(A,B)**. Scale bars: 250 μm **(A,B)**; 10 μm **(C–F)**.

GABAergic axons from the basal ganglia output nuclei (SNr and ENT) are prominent sources of input to the ventral motor thalamus. In addition, as all other nuclei of the thalamus, the motor nuclei receive GABAergic input from the reticular prethalamic nucleus (Hádinger et al., 2023). To gain insight on the spatial distribution of these important inputs in the ventral motor nuclei neuropil we immunostained for two specific markers of presynaptic vesicle pools in GABAergic axon terminals, the vesicular GABA transporter (vGAT) and the enzyme glutamate decarboxylase 65/67 (GAD65/67; Esclapez et al., 1994). The patterns produced by these two markers were identical, and we describe these observations together. Immunolabeling for vGAT or GAD65/67 was uneven across thalamic nuclei (Figures 2A, B), but the differences were not sharp. A higher magnification analysis revealed that large puncta were very abundant in VA and VM. In contrast, the VL neuropil contained mostly small puncta, but also some irregular patches of large puncta (Figures 2C–F).

### Labeling of the zona incerta and anterior pretectal nuclei projections to the ventral motor nuclei

The presence of the numerous large vGAT puncta in VA and VM is in register with the well-known innervation that these nuclei by the basal ganglia output nuclei SNr and ENT (Deniau et al., 1992; Sakai et al., 1998; Bodor et al., 2008; Kuramoto et al., 2011; Foster et al., 2021; see below).

**FIGURE 3:**
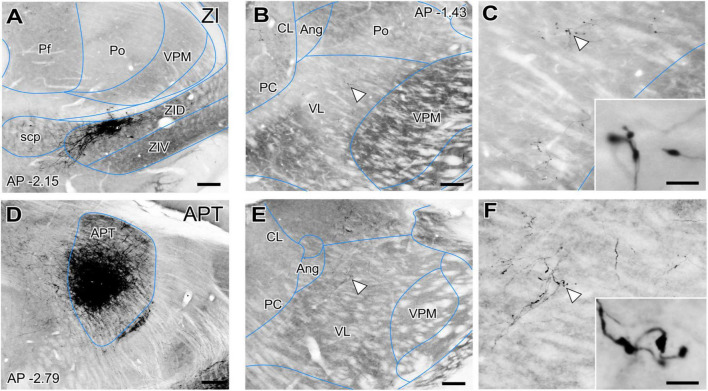
Non-basal ganglia sources of putative GABAergic afferents to the ventral thalamic motor nuclei. **(A)** Coronal section of the mouse thalamus showing representative BDA iontophoretic microinjection on the dorsal zona incerta (ZID). Cytochrome-C oxidase counterstain. **(B,C)** Coronal section of the thalamus showing a BDA-labeled varicose arborization from a ZID axon in VL. Panel **(C)** and its inset illustrate the same axon at higher magnifications. **(D)** Coronal section showing the center of a representative BDA iontophoretic microinjection in the anterior pretectal nucleus (APT). Cytochrome-C oxidase counterstain. **(E,F)** Coronal section of the thalamus showing a BDA-labeled varicose arborization from APT axons in VL. Panel **(F)** and its inset show the same axon at higher magnifications. White arrows points at labeled axons. Scale bars: 250 μm **(A–D)**, 200 μm **(B–E)**, 5 μm **(C–F)**.

However, the origin of the large GABAergic puncta in VL was unclear, as this nucleus is not known to be directly targeted by the basal ganglia output nuclei. GABAergic projections with large axonal boutons from the zona incerta (ZI, Power and Mitrofanis, 1999; Barthó et al., 2002) as well as from the anterior pretectal nucleus (APT; Bokor et al., 2005) have been reported in association and intralaminar thalamic nuclei. In rats some ZI terminals reach the VL nucleus (Barthó et al., 2002). Thus, we examined the anterograde labeling produced in the ventral motor nuclei of mice after BDA injections (Figures 3A–D). These experiments show that both ZI and APT axons form focal clusters of large terminal varicosities in VL (Figures 3B, C, E, F) and VM (not shown).

### Double-labeling analysis of the distribution of GABA and vGLUT2 immunolabeling

To directly elucidate the convergence/segregation of the neuropil territories targeted by subcortical glutamatergic afferents or GABAergic afferents we performed a double-immunofluorescence analysis with antibodies against vGLUT2 and vGAT/GAD65/67 on the same tissue sections.

**FIGURE 4:**
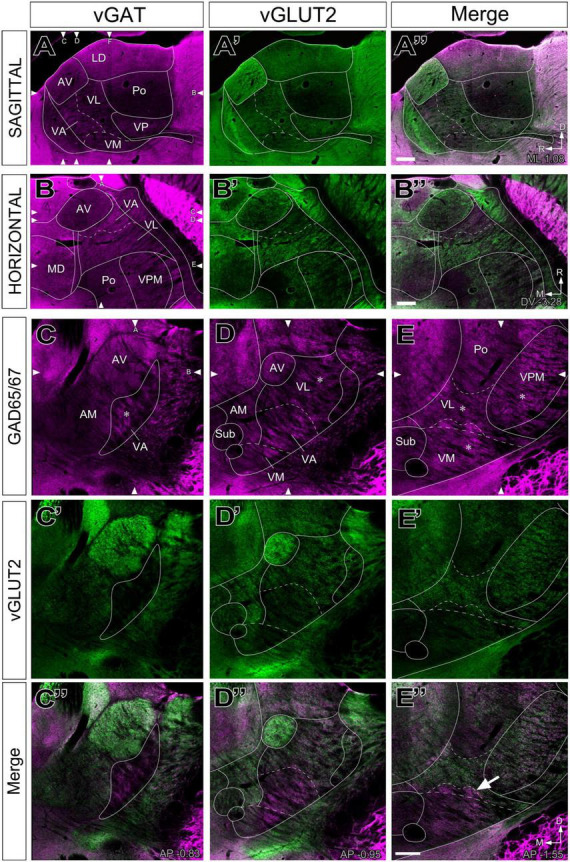
Double–fluorescence immunolabeling for glutamatergic (vGLUT2) and GABAergic (vGAT or GAD65/67) markers in the mouse ventral thalamic motor nuclei. Multichannel confocal imaging of thalamus sections fluorescently immunolabeled for vGAT (**A**; magenta), vGLUT2 (**A’**; green), and their merge **(A”)** in sagittal sections (dorsal is to the top and caudal is to the right) and in horizontal sections vGAT (**B**; magenta), vGLUT2 (**B’**; green), and their merge **(B”)**, rostral is to the top and lateral is to the right. And multichannel confocal imaging of coronal thalamus sections fluorescently immunolabeled for GAD65/67 (**C–E**; magenta), vGLUT2 (**C’–E’**; green), and their merge **(C”–E”)**. Dorsal is to the top and lateral to the right. For reference, in each sectional plane, arrowheads indicate the section levels at which the images shown in the other two orthogonal planes were taken. Bregma level in mm is indicated in the upper right or inferior right corner. Note that neuropil regions with less vGLUT2 expression are largely complementary to those enriched in vGAT or GAD65/67. The asterisks (*) in panels **(C–E)** indicate the approximate location of the high-magnification details in Figure 5. The arrow in **(E”)** points at the GABAergic territory found more dorsal of the cytoarchitectonic border of the VM. Scale bars: 250 μm **(A”,B”,E”)**.

The labeling patterns for each of the neurotransmission markers were fully consistent with the bright-field single antibody observations. In addition, this analysis revealed that vGLUT2 and the heavily GABA immunopositive territories in VA-VL are complementary (Figures 4A”–B”, C”, D”). Likewise, the VM neuropil is heavily labeled by the GABA neurotransmission markers except for its most lateral and caudal region, which is immunopositive for vGLUT2 (Figure 4E”).

**FIGURE 5:**
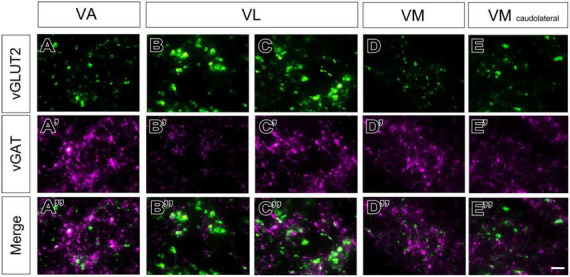
High-magnification detail of glutamatergic (vGLUT2) and GABAergic (vGAT) immunolabeled puncta in different ventral thalamic motor nuclei. Representative samples taken from different nuclei showing vGLUT2 (**A–E**; green), vGAT (**A’–E’**; magenta) or merged images of both **(A”–E”)**. **(B–C”)** For VL two separate samples are shown, one taken from a region with only small vGAT puncta (asterisk in Figure 4D), and other from a region with both large and small vGAT puncta (asterisk in Figure 4E). In addition to the ventral motor nuclei, a sample from the caudolateral portion of the ventromedial nucleus (VM caudolateral) is illustrated for comparison. The regions sampled are indicated by asterisks in Figure 4, **(C–E)**. Scale bar: 10 μm **(A–D”)**.

A higher magnification analysis revealed that the neuropil in the various nuclei contain specific combinations of immunolabeled puncta. In addition to numerous large vGAT puncta, the neuropil of VA and VM contained numerous smaller vGLUT2 puncta (Figures 5A–A”, D–D”). In contrast, the VL neuropil contained mostly large vGLUT2 puncta (Figures 5B–B”) and also some small vGAT puncta (Figures 5B’–B”). In addition, the lateral region of VL (Figure 4D), showed some irregular groups of large vGAT puncta (Figures 5C–C”). The caudolateral portion of VM contains large vGLUT2 puncta but lacks large vGAT/GAD65/67 puncta (Figures 5E–E”).

### Distribution of calbindin immunolabeling in the ventral motor nuclei

**FIGURE 6:**
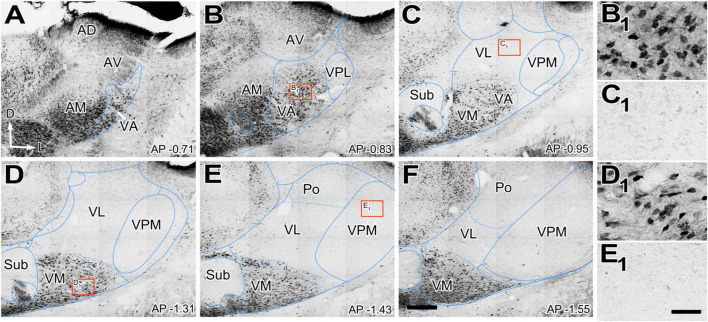
Distribution of calbindin (CALB1) immunolabeled cells in the ventral thalamic motor nuclei. **(A–F)** Images showing in different coronal sections of VA, VL, and VM the distribution of terminals marked by immunohistochemistry against calbindin, (CALB1). Dorsal is to the top and lateral to the right. High-magnification details showing calbindin positive labeled somas in the rostral pole of the VA-VL complex, VA **(B_1_)** and in VM **(D_1_)**. Note the absence of positive labeled somas in the dorsal and caudal part of the VA-VL complex **(C_1_)** and the absence in VPM **(E_1_)**. Bregma level in mm is indicated in the inferior left corner. Scale bars: 250 μm **(A–F)** and 25 μm **(B_1_,C_1_,D_1_,E_1_)**.

Calbindin type 1 28kD (CALB1) has been frequently used as a general marker for delineating functional compartments within thalamic nuclei (Jones, 2007; Kuramoto et al., 2009, 2011). Thus, we immunostained for this marker in mouse tissue sections (Figure 6). We found that CALB1 expression in the ventral nuclei largely correlates with heavy GABA neurotransmission marker labeling. Large numbers of CALB1-positive cell somata (Figures 6B1, D1) were located in the same territories observed to contain large puncta immunopositive for GABA neurotransmission markers (compare Figure 5 with Figures 6C1–E1). Again, a continuous population of heavily labeled cell somata extended all over VA and VM. In contrast, VL was essentially free of immunopositive neurons. Interestingly, the positive calbindin labeled cells extended for few hundred microns dorsally to the traditional cytoarchitectonic border of VM. However, the caudolateral corner of the VM was as heavily stained as the rest of this nucleus.

Overall, the multi labeling analysis of the ventral motor nuclei reveals diverse combinations of large or small glutamatergic or GABAergic inputs across the nuclei neuropil. Such differences imply that thalamic neurons in different portions of these nuclei receive specific combinations of subcortical excitatory and/or inhibitory inputs.

### Distribution of cerebellar afferents in the ventral motor nuclei

The observed differences in glutamatergic and GABAergic puncta might result from the presence/absence of different afferent systems and/or from variation in the size of the synaptic terminals in different branches of the same input axons (Sidibé et al., 1997; Viaene et al., 2011; Rodriguez-Moreno et al., 2020). To directly investigate the distribution and terminal axon morphology of the main input systems innervating the ventral motor nuclei, we examined anterograde tracing experiments involving injections of AAV vectors driving the expression of fluorescent proteins (see “Materials and Methods”) in the output nuclei of the cerebellum or basal ganglia.

**FIGURE 7:**
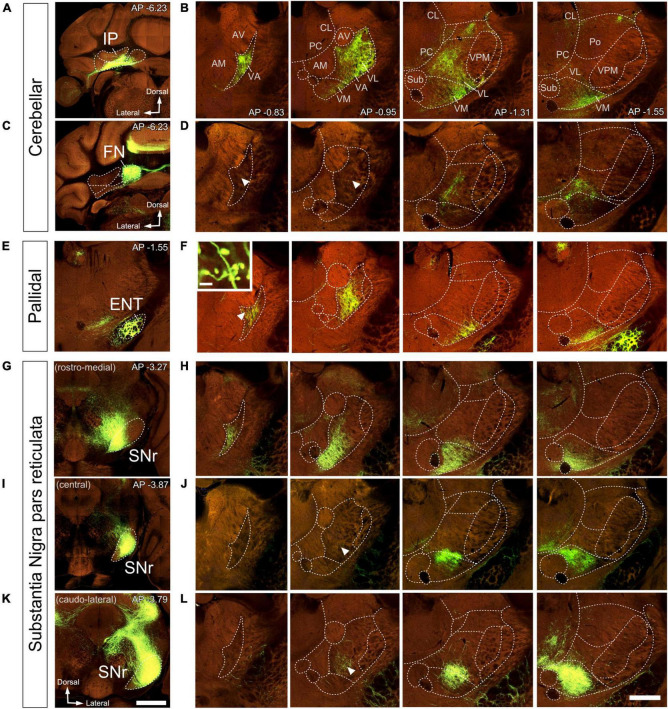
Anterograde labeling of cerebellar, pallidal and nigral inputs to the ventral thalamic motor nuclei by viral vector-mediated fluorescent protein transfection. Two-photon tomography image samples in representative bregma level sections. Injection sites in IP, **(A)**, FN **(C)**, ENT **(E)**, or in three different domains of SNr **(G–K)**. **(B,D,F,H,J,L)** Coronal section images showing axons labeled (green fluorescence) in the ventral motor nuclei in each injection case. In addition to the evident dense plexuses of labeled fibers, sparser axons are present in some regions (indicated by arrowheads). The inset in **(F)** shows big size varicosities in VA arising from the ENT. Images from the Mouse Connectivity Projection dataset https://connectivity.brain-map.org/projection/experiment/304474221 **(A,B)**,/127650431 **(C,D)**,/305024724 **(E,F)**,/158914182 **(G,H)**,/175263063 **(I,J),** and /299895444 **(K,L)**. Bregma level (in mm) is indicated in the upper right **(A–K)** or inferior right **(B)** corner. Scale bars: 1 mm (injections, **A,K**) and 500 μm **(B,D,F,H,J,L)**, 35 μm (**F** inset).

**FIGURE 8:**
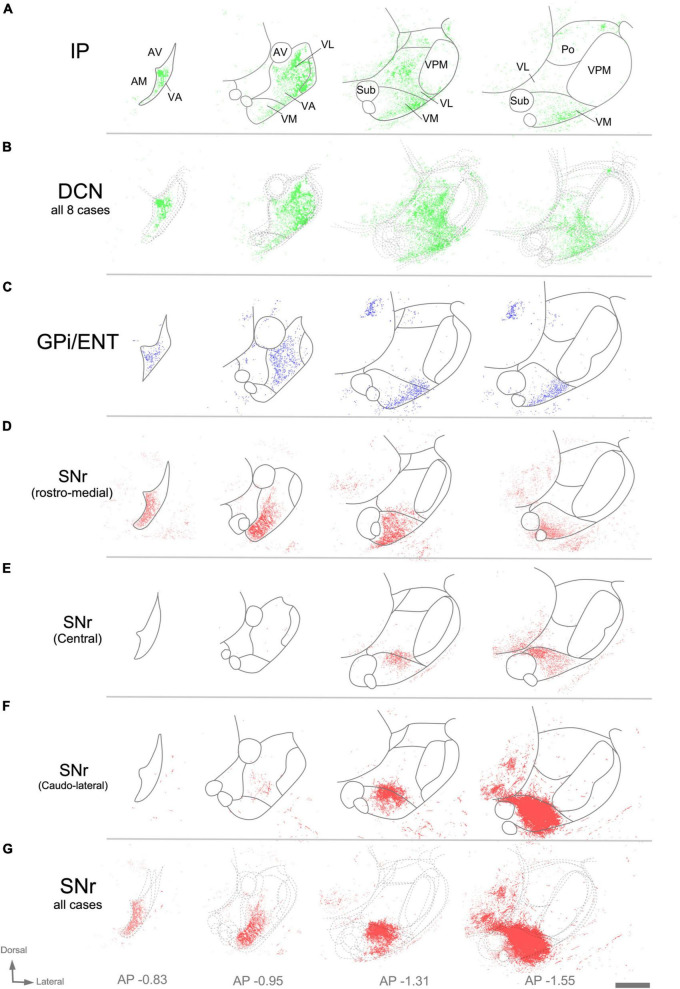
Image analysis of labeled axon varicosities in fluorescent anterograde tracing experiments. Axonal varicosities in the image datasets were segmented using the supervised machine-learning (bio) image analysis software Ilastik. Panels **(A,C,D,E,F)** illustrate individual experiments (compare with Figure 7), while **(B,G)** are cumulative overlays on multiple cases (DCN, *n* = 8; SNr, *n* = 3) from Mouse Connectivity Projection datasets to generate a more global view of the afferents from the various cerebellar nuclei **(B)** or the SNr **(G)**. Other conventions as in Figure 7.

Vector injections in the DCN (Figures 7A–D, 8A, B), labeled abundant terminal arborizations that were unevenly spread across the ventral motor thalamus. Remarkably, the arborizations labeled from injections in different cerebellar nuclei overlapped widely within the thalamus, showing limited segregation or topographical order. Figures 7A, B shows an example of a AAV injection in the interposed cerebellar nucleus (IP) and Figures 7C, D shows an example of an injection in the fastigial cerebellar nucleus (FN). Following these two injections, labeled cerebello-thalamic axon arborizations were most profuse in the VA-VL nucleus and the lateral half of VM. The axonal arborizations extended up to the rostralmost end of VA-VL, (Figure 8A). A band about 150 μm wide along the medial border of the VA-VL nucleus and the most of the medial part of VM were virtually free of cerebellar terminals in this and the rest of DCN injection experiments. In addition to ventral nuclei, sparse terminal arborizations were observed in other thalamic nuclei of the thalamus such as the central lateral and paracentral intralaminar, in the lateral portion of the mediodorsal (Figures 7B–D), and in the parafascicular (not shown).

Within thalamic projection axon arborizations, varicosities are the primary location of synapses (Rodriguez-Moreno et al., 2020). To selectively visualize varicosities without interference from the fluorescence of non-varicose axon segments, we trained an artificial intelligence segmentation algorithm (see “Materials and Methods”) and applied it onto high-magnification images of the AAV vector labeling. This analysis (Figure 8) revealed that the cerebellar innervation is markedly uneven, as it concentrates in several patches located in central and lateral portions of VL while leaving other zones of this nucleus much less densely innervated (Figure 8B).

### Distribution pallidal afferents in the ventral motor nuclei

In rodents, the GPi is a relatively small group of cells dispersed among the fibers of the internal capsule and also known as ENT. An AAV injection selectively involving the medial and caudal part of GPi/ENT (Figure 7E and Supplementary Figure 2A) selectively labeled a dense plexus of axon arborizations in the most rostral zone of VA-VL (Figure 7F and Supplementary Figure 2B), while sparing the central and caudal portions of the nucleus. A second focus of pallidal innervation was present in the lateral and ventral portion of VM, the territory through which the GPi/ENT axons enter the thalamus. The varicosity segmentation analysis showed that this territory contains numerous varicosities, in addition to the trunks of the pallidal axons (Figure 8C). A small patch of labeling was visible in the lateral habenula, and few fibers were labeled in the parafascicular nucleus (Supplementary Figures 2C, D). The rest of the thalamus was free of labeling.

In striking contrast, an injection selectively involving the anterior and lateral portion of GPi/ENT did not label any axon in VA-VL but labeled a profuse plexus of axons in the parafascicular nucleus in the lateral habenula (Supplementary Figures 2E–H).

### Distribution of nigral afferents in the ventral motor nuclei

Vector injections in the Substantia Nigra pars reticulata (Figures 7G–L) labeled dense terminal arborizations plexuses in VM and VA, but almost completely spared VL. Interestingly, the nigral arborizations were in every experiment found to extend for some distance beyond the traditional dorsal border VM.

**FIGURE 9:**
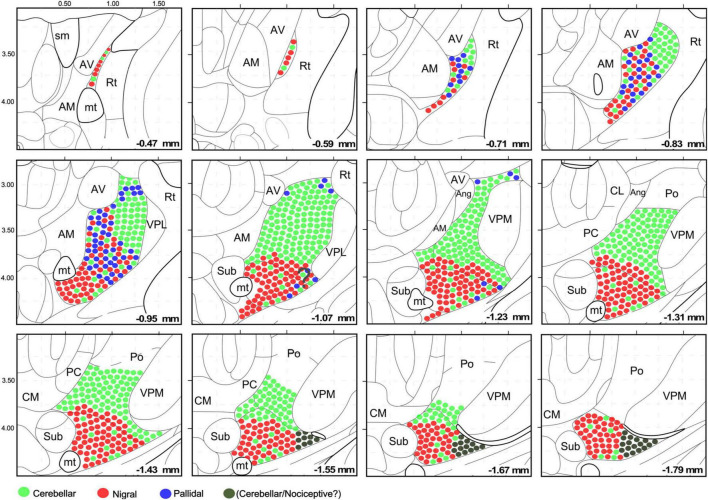
Schematic representation of the cerebellar, pallidal and nigral input landmark on top of the Paxinos and Franklin’s (2019) mouse brain delineation. The distribution and intermingling of different input classes are schematically indicated by circles of different colors. All circles are the same size; differences in bouton size are not represented for clarity. Note that the cerebellar territory is highly segregated, while the nigral and pallidal territories overlap extensively. This representation takes into consideration the chemoarchitectonic and input connections derived from this study.

Unlike the AAV injections in the various DCN, injections in different SNr regions labeled each axon arborization in a specific region. An injection in rostromedial (Figures 7G, H, 8D) SNr labeled arborizations across the whole rostrocaudal extent of VM and VA, from its very anterior tip (level −0.47 mm in Figure 9; see also Figure 7H) to the caudal region of the nucleus where VM cell somata begin to disappear amidst the fibers of the superior cerebellar peduncle (level −1.79 mm; Supplementary Figure 3 and Figure 9). In contrast, injections in the central or caudolateral SNr labeled arborizations spared the rostral pole of in the caudal half of VM (Figures 7I–L, 8F, G; bregma levels −1.55 to −1.79 mm).

### Axonal varicosity sizes of the cerebello-thalamic pathway

**FIGURE 10:**
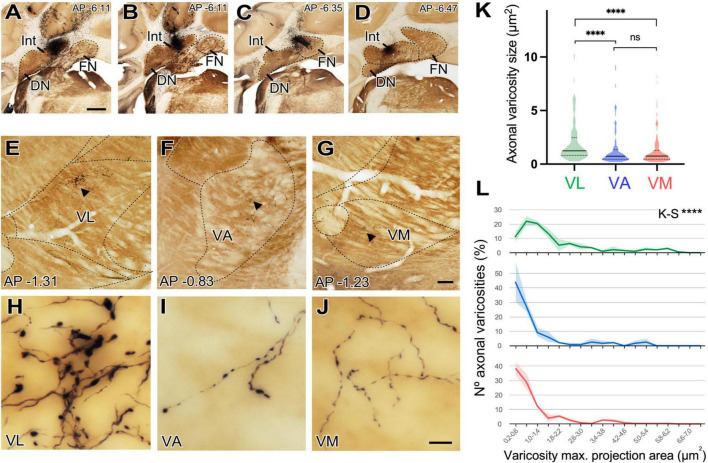
Size differences of cerebellar axon varicosities in different ventral thalamic motor nuclei. **(A–D)** Examples of a BDA deposit in the interpositus **(A,B)** or fastigial **(C,D)** deep cerebellar nuclei. Cytochrome C-oxidase counterstain. Dorsal is to the top and medial to the right. Coronal level posterior (-) to Bregma, in mm, is indicated on the upper right corner. **(E–G)** Coronal section images of the ventral motor nuclei of the contralateral thalamus showing BDA-labeled axonal arborizations in VL (panel “**E**”), VA (“**F**”) or VM (“**G**”). Cytochrome C-oxidase counterstain. **(H–J)** High-magnification detail of the varicose axon branches shown in panels **(E, F)**. **(E–J)** Dorsal is to the top and medial is to the right. Scale bars: **A–D** = 1,000 μm, **E–G** = 100 μm, and **H–J** = 10 μm. **(K,L)** Quantitative comparisons of cerebellar axon varicosity sizes in VA, VL and VM. Maximal projection area (in μm^2^) was measured as a proxy for bouton size. Panel **(K)** shows a violin-plot charts showing density distribution of the data. Median is indicated by a continuous black line and quartiles by dotted lines. Statistical differences were computed using the Kruskal–Wallis test followed by the Dunn’s *post-hoc* test. Panel **(L)** shows the frequencies distribution of axon varicosity sizes. Values represent percentage of mean ± SEM (paler shadings of the same colors). For each nucleus data were plotted from *n* = 2 BDA labeling experiments. The Kolmogorov–Smirnov test was used for comparisons of varicosity size distributions. ****p < 0.001.

Deep nuclei of the cerebellum (DCN) projections are known to be the main or exclusive glutamatergic subcortical input to the motor thalamus. Because we had observed both that DCN axons reach all the ventral motor thalamus (Figure 8B) nuclei and that these nuclei contain large and small vGLUT2 immunopositive puncta (putative glutamatergic vesicle pools) (Figures 5A”, B”, D”) we decided to test if cerebellar axon varicosities have different sizes in the various motor thalamus nuclei. To this end, we selectively labeled small populations of cerebello-thalamic projection axons by means of microiontophoretic BDA injections in the DCN (Figures 10A–D) and compared their varicosity sizes.

Axons labeled from injections in different DCN always entered the thalamus to the superior cerebellar peduncle and arborize to target separate thalamic territories (Figures 10E–G). The morphology of the terminal arborizations was strikingly different between those branches situated in VL and those localized in VA or VM (Figures 10H, I, J). In VL, cerebellar formed clusters of terminal branches with large varicosities (putative synaptic boutons), many of which were situated as a bulbous head at the tip of a small branch (Figure 10H). In contrast, cerebellar axon branches situated in VM or VA displayed more linear patterns and their varicosities were markedly smaller. To confirm this impression, we measured varicosity size (maximal projection area) and compared it across nuclei. Median size in VL was 1.24 μm^2^, but only 0.74 μm^2^ and VM 0.76 μm^2^ for those in VA and VM and this difference was significant (*p* < 0.0001). No significant differences were found between the axonal varicosities that ended in the VA and those that ended in the VM. The median sizes are represented in a violin plot (Figure 10K). The varicosities of VA and VM were both similar between them and had a significantly different frequency from those of VL (K–S, *p* < 0.0001; Figure 10L). Statistical data are summarized in Table 2.

**TABLE 2:** Quantitative analysis of axonal varicosity sizes from the cerebellum.

	Median (μ m^2^)	Test			
	–	–	VL		
VL	1.24	Dunn	–		
		K–S	–	VA	
VA	0.74	Dunn	0.000****	–	
		K–S	0.000****	–	VM
VM	0.76	Dunn	0.000****	0.864	–
		K–S	0.000****	0.957	–

Comparison of varicosity median cross-sectional areas was performed using the Kruskal–Wallis test followed by the Dunn’s post-hoc test for multiple comparison. Kolmogorov–Smirnov test (K–S) was used to compare between different structures the size distribution of varicosities. ****p < 0.0001.

## Discussion

We provide the first systematic mapping of cerebellar and basal ganglia inputs to the mouse ventral motor nuclei. To this end, we analyzed the distribution of glutamate and GABA vesicular transporters in the neuropil, and mapped the territories targeted by axons originated from either the deep cerebellar nuclei (DCN), substantia nigra (SNr), or entopeduncular nucleus/internal globus pallidus (ENT/GPi). Together, these data reveal input-specific combinations that delineate three main thalamic territories. The re-defined territories are relevant for both experimental and modeling investigations of the rodent motor system, as well as for comparison with other species.

### Input heterogeneity in the ventral motor nuclei neuropil

**FIGURE 11:**
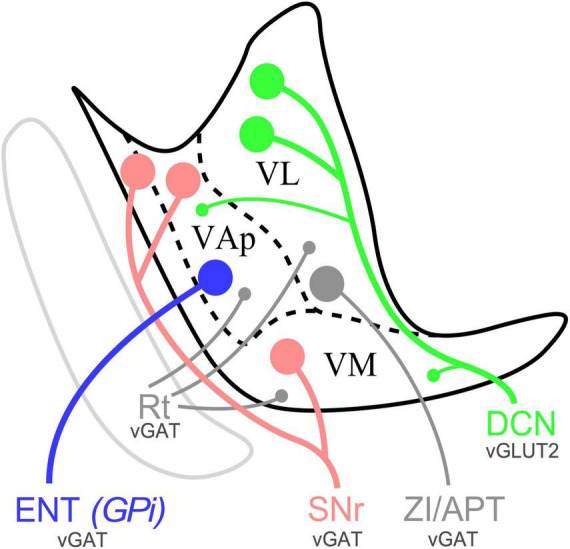
Schematic distribution of SNr, ENT/GPi and DCN inputs in the ventral motor thalamus of mice and representative varicosity size. Sagittal section diagram of the mouse ventral motor nuclei. Dots represent varicosity sizes, big or small. VL, ventral lateral nucleus; VAp, pallidal ventral anterior nucleus; VM, ventromedial thalamic nucleus; Rt, reticular nucleus; ZI, zona incerta; APT, anterior pretectal nucleus.

Our immunolabeling for presynaptic markers of subcortical glutamatergic or GABAergic origin revealed diverse combinations of putative input synapses across the ventral motor nuclei (Figure 11; Casas-Torremocha et al., 2022). Moreover, differences in the size of immunolabeled puncta suggest different presynaptic neurotransmitter vesicle pools sizes (Esclapez et al., 1994; Bopp et al., 2017).

In the dorsocaudal portion of the thalamic territory usually referred to as VA-VL [Paxinos and Franklin, 2019; also identified as “ventral anterior-lateral complex” (VAL; Wang et al., 2020)] the neuropil is characterized by the presence of abundant large glutamatergic vGLUT2 and numerous small GABAergic puncta (Figures 4, 5). Conversely, the rostroventral portion of VA-VL is characterized by abundant large GABAergic puncta but only small vGLUT2 puncta. An equivalent complementary pattern was described in rat VA-VL by Kuramoto et al. (2009, 2011), which they called excitatory and inhibitory afferent-dominant zones, respectively.

In addition, the neuropil in most of the territory traditionally referred to as “ventromedial nucleus” (VM; Rioch, 1929; Jones, 2007; Paxinos and Franklin, 2019, Wang et al., 2020) displays the same immunolabeling pattern present in rostroventral VA-VL without any hint of a border between the two (Figures 4D, D”). The same pattern extends dorsally across the cytoarchitectonic dorsal border of VM for some distance (arrow in Figure 4E”, see also Figures 1, 4, 6).

In contrast, the most caudal and lateral portion of the traditional VM nucleus is largely devoid of GABAergic puncta yet, unlike the rest of VM, it contains large vGLUT2 puncta (Figures 1G, H, G1, 5E–E”). A possible source for these puncta may be a brainstem nociceptive pathway (Figure 9; Villanueva et al., 1998). Since the cerebello-thalamic axons enter the thalamus precisely through this zone, we cannot exclude the possibility that they also leave some boutons there (Figure 7B).

The schematic representation of the cerebellar, pallidal and nigral inputs that we present in Figure 9 may help the accurate interpretation of connectomic studies of the mouse motor cortex, cerebellum and basal ganglia.

### Territories targeted by the DCN and BG pathways

The deep cerebellar nuclei project di-synaptically to the motor cortex through the ventral thalamus (Allen and Tsukahara, 1974; Kelly and Strick, 2003). We show that the dorsocaudal part of VA-VL and VM are targeted by DCN axons, in line with previous observations in rats (Faull and Carman, 1978; Angaut et al., 1985; Deniau et al., 1992; Aumann et al., 1994; Kuramoto et al., 2011). In addition, we show that the cerebellar axons form clusters of large terminal varicosities in the dorsocaudal VA-VL but are not clustered and have only small varicosities in VM. Notably, the cerebellar axons also reach the anteromedial portion of VA-VL, a region not usually regarded as a cerebellar target. As in VM, the axon varicosities here are small, and do not form clusters.

The entopeduncular nucleus is a key output channel of the basal ganglia system. It projects downstream to the lateral habenula and the pedunculopontine tegmental nucleus, but also signals back to the cortex via its projections to the ventral motor nuclei (Kuo and Carpenter, 1973; Kim et al., 1976; Larsen and McBride, 1979). The mouse ENT/GPi consists of cell islands scattered among the fibers of the internal capsule. For this reason, we did not try to label the pallidothalamic axons with BDA, and instead analyzed AAV injection data, as AAV vectors do not usually transfect myelinated axons (Porrero et al., 2016; Kuramoto, 2019). We examined several AAV deposits that together covered the whole extent to ENT/GPi. These data reveal a highly compartmentalized organization of the ENT/GPi output pathways, consistent with observations in rats (Van Der Kooy and Carter, 1981; Kalen et al., 1989; Rajakumar et al., 1993; Takada et al., 1994; Kha et al., 2000) and primates (Hendry et al., 1979; Ilinsky and Kultas-Ilinsky, 1987; Percheron et al., 1996; Jones, 2007). It is of note that the pallidal projection to the ventral motor nuclei originates exclusively from a caudomedial portion of ENT/GPi and targets a small territory in the rostroventral portion of VA-VL.

The SNr is the main subcortical input to rodent VM (Deniau et al., 1992; Cebrián et al., 2005; Bodor et al., 2008; Kuramoto et al., 2015). Our data show that, in mice, the SNr axons enter through the caudal pole of VM and arborize densely in the nucleus. Moreover, the arborizations extend into the rostroventral portion of VA-VL, while sparing more dorsocaudal VA-VL portions. The arborizations also extend for about 100 μm dorsally to the traditional dorsal border of “VM” (Figure 8F; 1.31 mm bregma level); this is a region found to lack large vGLUT2 puncta and to contain large vGAT/GAD65/67-positive puncta (Figures 1, 4, 6). The overall implication is that this zone may be functionally regarded as part of “VM,” despite cytoarchitectonic appearances. Consistent with observations in cats and rats (Kemel et al., 1988; Deniau et al., 1992), we show that the mouse SNr-thalamic projection displays a substantial level of topographic organization consistent with the overall highly parallel and modular organization of the basal ganglia circuitry [reviewed in Haber (2003)].

### Specific combinations of inputs define functional territories

While consistent with previous studies indicating a substantial convergence of DCN and BG inputs in the ventral motor nuclei of the rodent thalamus (Faull and Carman, 1978; Angaut et al., 1985; Deniau et al., 1992; Kuramoto et al., 2011), our observations reveal several distinct input combinations across the ventral motor region of the mouse thalamus. Differences involve the input sources, their neurotransmitters and their bouton/puncta sizes (Figure 11). Note that bouton size differences are relevant as they imply variation in strength and dynamic response properties (Rodriguez-Moreno et al., 2020; Acsády, 2022). The overall implication is that the neurons situated in each of the territories defined by the input combinations are bound to perform fundamentally different computations (Acsády, 2022).

The neuropil in a large territory of the dorsocaudal part of classic “VA-VL” is dominated by cerebellar inputs (Figures 8B, 10H), which terminate as clusters of large varicosities, and are the source of the large vGLUT2 puncta (Kuramoto et al., 2011). It also contains small vGAT/GAD65/67 puncta probably arising from the reticular prethalamic nucleus (Hádinger et al., 2023). Interestingly, we also found a scattered population of large vGAT in the lateral part of the nucleus. Our anterograde tracing data in mice (Figure 3) along with previous evidence in rats from other authors (Power and Mitrofanis, 1999; Barthó et al., 2002; Bokor et al., 2005) strongly suggest that these puncta correspond to axons from the zona incerta and/or APT. We propose to retain the name ventral lateral nucleus (VL) exclusively for this territory.

A small rostroventral territory (AP bregma levels −0.71 to −0.95 mm) contains small vGLUT2 and large vGAT/GAD65/67 puncta in its neuropil. This region receives richly branched axons from the medial part of the ENT/GPi and from the SNr, both of which form large terminal boutons (Figure 7F; Bodor et al., 2008) and are thus the likely sources for the vGAT/GAD65/67 puncta. Cerebellar axons with small en passant varicosities also target this region and are the likely source of its small vGLUT2 puncta. We label this territory as pallidal ventral anterior nucleus (VAp).

Most of the classic “VM” nucleus contains small vGLUT2 puncta and large vGAT/GAD65/67 puncta. The equivalent territory in rats receives heavy innervation from the SNr, with large GABAergic boutons (Bodor et al., 2008), as well as cerebellar axons with small varicosities (Figure 10J). Previous studies in cats (Hendry et al., 1979; Noda and Oka, 1985; Steriade, 1995) and rats (Faull and Carman, 1978; Angaut et al., 1985; Aumann et al., 1994) noted a comparable overlap between the cerebellar and basal ganglia outputs in “VM.” We propose to retain the term ventromedial thalamic nucleus (VM) exclusively for this territory.

It is of note that the neuropil in the most caudal and lateral portion of the classic “VM” nucleus contains large vGLUT2 puncta but lacks large vGAT/GAD65/67 puncta. Direct afferents from a brainstem nociceptive pathway may be the source of glutamatergic puncta (Villanueva et al., 1998). Cerebellar axons may contribute additional vGLUT2 puncta to this region. For these reasons, we propose to distinguish this zone from the rest of the classic “VM” as the lateral ventromedial nucleus (VMl). The markedly different presynaptic varicosity sizes of the cerebellar afferents terminating in VL compared to those in VAp and VM suggest that they may have different synaptic dynamic profiles and impact on the cells in these nuclei. In glutamatergic axons, larger axonal varicosity sizes have been linked to more effective synaptic transmission (Sherman and Guillery, 2002; Mukherjee et al., 2020). Besides, our data indicate that in mice SNr, pallidal and DCN signals can converge onto VAp and VM cells. Future transsynaptic labeling and/or electron-microscopic strategies could further clarify this point. This is in contrast with the situation in primate thalamus where the three pathways remain largely separate, and convergence occurs primarily in the cortex (Haber, 2003; Barbas et al., 2013; see below).

Interestingly, our input-defined neuropil territories show a partial correlation with the distribution of two high-level projection cell classes recently identified in the mouse thalamus based on morphological, electrophysiological and gene expression data (Figure 9; Clascá, 2022). The VL neuropil territory is populated by “multifocal” projection neurons. These neurons are characterized by their profuse second-order dendrites, and by having axons that arborize focally in the intermediate layers of the motor and somatosensory cortices but do not arborize in the striatum (Kuramoto et al., 2009, 2015). In contrast, the neurons located in the VAp and VM neuropil territory are of the “subpial projection” type. Such cells have relatively fewer second order dendrites, and their axons innervate both the striatum and the cerebral cortex. In the cortex they arborize preferentially in layer 1, often extending over large territories. Moreover, these two high-level cell types show consistent differences in their electrophysiological profile and can be separated based on their transcriptomic profile (Phillips et al., 2019). Consistent with the immunostaining patterns (Figure 6; see also Kuramoto et al., 2009, 2011). Calbindin is one of the genes that shows a clear difference in their level of expression between the nigral-recipient and cerebellar recipient territories (Phillips et al., 2019).

### Comparison with input-based nuclei delineations in the macaque motor thalamus

Computations carried out by the thalamic neurons are fundamentally determined by the source(s) and the spatial distribution and dynamic properties of the input synapses (Halassa and Sherman, 2019; Acsády, 2022). Unlike cytoarchitectonic appearance or other classic neurohistological criteria, input-based nuclear divisions are thus directly related to function. Moreover, they could be consistently comparable across species (Percheron et al., 1996; Jones, 2007). Therefore, our observations in mice are relevant for comparisons with the primate thalamus.

**FIGURE 12:**
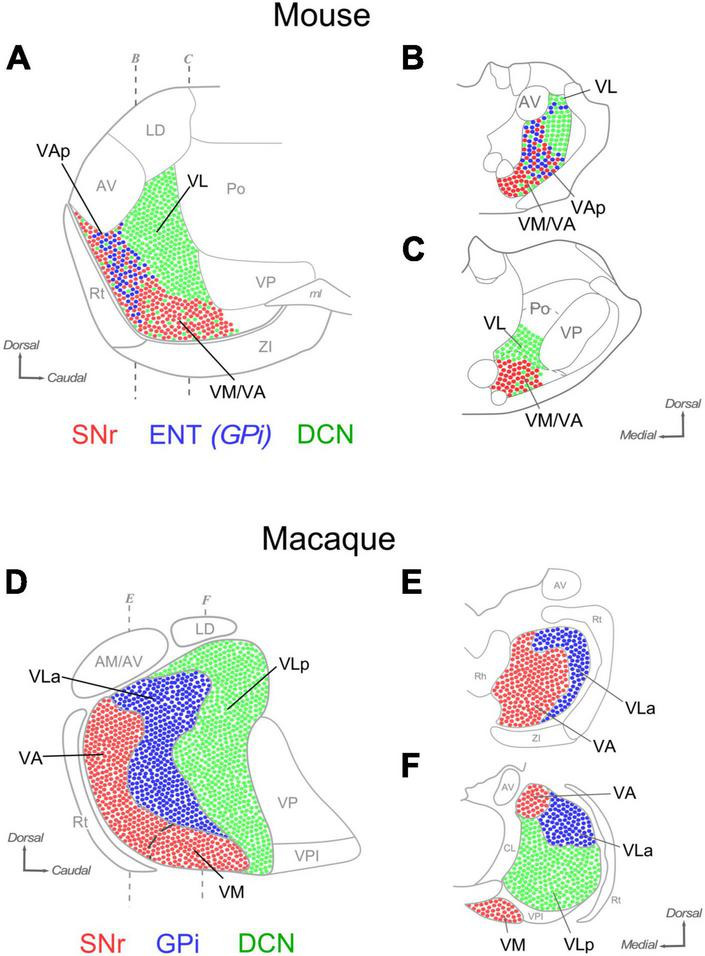
Comparison of the distribution of SNr, ENT/GPi and DCN inputs in the ventral motor thalamus of mice and macaques. Sagittal **(A)** and coronal **(B,C)** section diagrams of the mouse ventral motor nuclei. Dots are used to represent in highly schematic form the distribution of axonal terminals originating in the DCN (green), GPi (blue) and SNr (red). Three main nuclear domains can be delineated based on input combinations **(A)**. Coronal sections of A **(B,C)**. **(D–F)** A highly schematic representation of the distribution of DCN, GPi and SNr axon terminals in the macaque ventral motor nuclei is depicted on diagrams of a sagittal section (panel “**D**”) and two coronal sections of the thalamus (panels “**E, F**”). Based on Jones (2007) and Percheron et al. (1996).

Despite a plethora of often conflicting nomenclatures (reviews in Percheron et al., 1996; Jones, 2007) three main segregated territories have been identified in the primate thalamus based on their input from the cerebellum, SNr or GPi, respectively (Figures 12D–F; Ilinsky and Kultas-Ilinsky, 1987; Percheron et al., 1996; Haber, 2003; Jones, 2007; Barbas et al., 2013).

The primate territory innervated by the cerebellar axons is referred to as “ventrolateral posterior” nucleus (VLp). The tag “posterior” distinguishes it from the pallidum-innervated territory (ventrolateral anterior nucleus; “VLa”; see below). For consistency with the primate, we thus propose to apply the same nomenclature (VLp, or VL, for brevity) to the mouse territory that is innervated by the cerebellum with large boutons and lacks inputs from the SNr or ENT/GPi (Figures 12A–C).

The territory innervated by the SNr axons in the ventral thalamus in macaques comprises two nuclei, the ventromedial (VM; Jones, 2007) and the ventral anterior nucleus, medial part (VAM; Paxinos et al., 2000). The two nuclei are distinguished by relatively minor cytoarchitectonic differences. In fact, some authors have considered them as a single nucleus (Percheron et al., 1996). We show that in mice the nigral territory involves the nuclei traditionally labeled as “VM” and an anterior portion of the “VA-VL” complex. In both species, the ventromedial thalamic nucleus (VM) denomination is applied to the region that receives predominant (mouse) or exclusive (macaque) nigral inputs.

In the large thalamus of macaques, the non-VM portion of the nigral territory and the pallidal territory are segregated, respectively, into two distinct nuclei: the “ventral anterior nucleus, medial part” (VAM) and the “ventrolateral anterior” (VLa) nuclei (Paxinos et al., 2000; Jones, 2007). In contrast, our data show that in mice the territory targeted by pallidal inputs is small and overlaps with nigral inputs. The pallidal thalamic territory zone had not been directly mapped in rodents. We propose to name it pallidal ventral anterior nucleus (VAp), or VAL, as in macaques.

In macaques, the SNr input territory wraps the most anterior tip of the ventral thalamic region, a territory labeled VA/VAM. The data presented here hint at the existence of a thin lamina of tissue at the rostral tip of the mouse ventral nuclei that is targeted by SNr axons, but not by ENT/GPi axons. Neuropil composition of this lamina is essentially the same as that of VM. In sum, subcortical input patterns demarcate three main territories, each characterized by a unique combination of inputs from the cerebellum, and/or the pallidum and/or the substantia nigra reticulata. Based on these patterns, we propose a new delineation of the mouse ventral motor nuclei that bears intriguing resemblances, and differences, with the organization of the macaque motor thalamus.

## Data availability statement

The original contributions presented in this study are included in this article/Supplementary material, further inquiries can be directed to the corresponding authors.

## Ethics statement

The animal study was approved by the Autonoma University Ethics Committee; Consejeria de Agricultura y Ganaderia, Comunidad de Madrid Regional Government (PROEX175/16 and PROEX179.3/21), in accordance with the European Community Council Directive 2010/63/UE. The study was conducted in accordance with the local legislation and institutional requirements.

## Author contributions

CA-M, CP, and FC: conceptualization, writing-original draft preparation, writing-review, and editing. CA-M: methodology and surgeries, investigation and reviewed literature, and prepared figures. CA-M and MR-T: immunolabeling and formal analysis. CP and FC: reviewed figures and resources funding acquisition. All authors contributed to the article and approved the submitted version.

## Footnotes

1
https://connectivity.brain-map.org/projection/experiment/experiment_id

2
www.graphpad.com
