# Corrigendum: Cerebellar and basal ganglia inputs define three main nuclei in the mouse ventral motor thalamus

**DOI:** 10.3389/fnana.2023.1301403

**Published:** 2023-11-22

**Authors:** Carmen Alonso-Martínez, Mario Rubio-Teves, Diana Casas-Torremocha, César Porrero, Francisco Clascá

**Affiliations:** Department of Anatomy and Neuroscience, Universidad Autónoma de Madrid, Madrid, Spain

**Keywords:** ventral anterior thalamic nucleus, ventral lateral thalamic nucleus, ventromedial thalamic nucleus, internal globus pallidus, substantia nigra pars reticulata, vesicular glutamate transporters, vesicular GABA transporter, cerebellum

In the published article, there was an error in the author list, and author Diana Casas-Torremocha was erroneously excluded. The corrected author list appears below.

Carmen Alonso-Martínez, Mario Rubio-Teves, Diana Casas-Torremocha, César Porrero and Francisco Clascá

The authors apologize for this error and state that this does not change the scientific conclusions of the article in any way. The original article has been updated.

