# Supplementary material for: Cerebellar and basal ganglia inputs define three main nuclei in the mouse ventral motor thalamus
Source: Front Neuroanat. 2023 Aug 14;17:1242839. doi: 10.3389/fnana.2023.1242839 (PMC10461449; doi:10.3389/fnana.2023.1242839)
Supplement: Supplementary file 1 [file Data_Sheet_1.pdf]

Supplementary Materials

Cerebellar and basal ganglia inputs define three main nuclei in the mouse ventral motor thalamus

Carmen Alonso-Martínez, Mario Rubio-Teves, César Porrero*, Francisco Clascá*

Department of Anatomy & Neuroscience,
Autónoma de Madrid University
Madrid 28029, Spain

*** Correspondence:** Francisco Clascá and César Porrero

francisco.clasca@uam.es

cesar.porrero@uam.es

1.1 Supplementary Figures

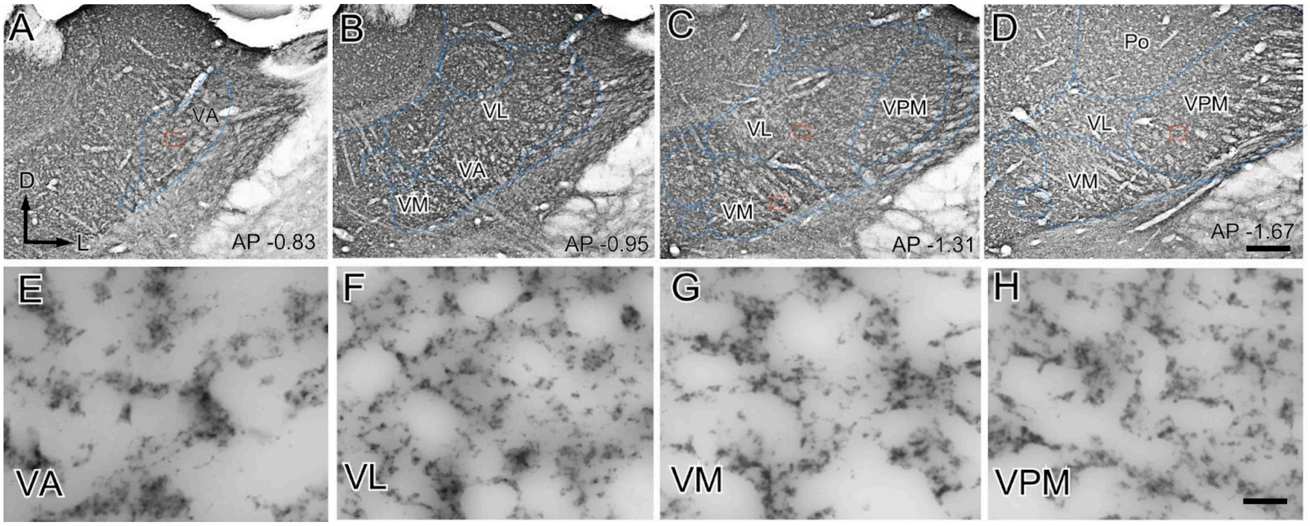

Supplementary Figure 1. Immunolabeling for vesicular glutamate transporter type 1 (vGLUT1).

A-D: Coronal photomicrographs. The immunolabeling did not reveal clear inhomogeneities between nuclei. Bregma level in mm is indicated in the inferior right corner. High-magnification details of the neuropil puncta in VA (**E**), VL, (**F**), VM (**G**) and VPM (**H**). Note that vGLUT1 puncta sizes puncta sizes are similar in different nuclei. Scale bars: 250 mm (**A–D**) and 10 mm (**E-H**).

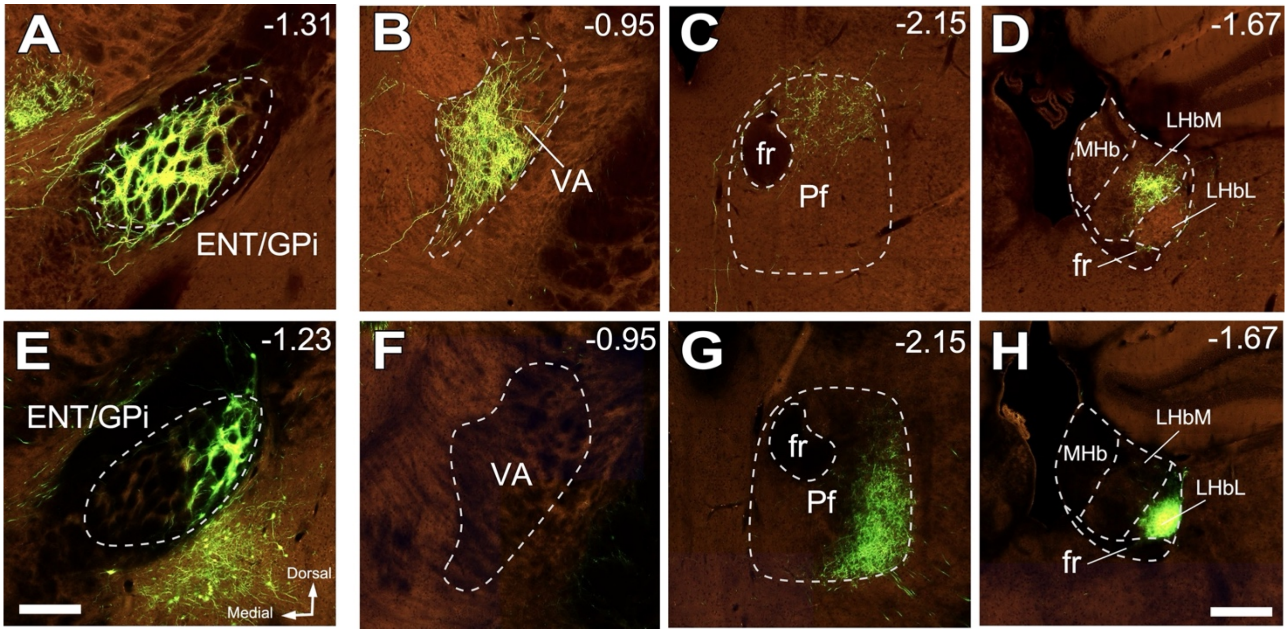

Supplementary Figure 2. Different regions of the GPi/ENT innervate markedly different territories of the thalamus and habenula.

(A and E), Coronal section image at the center of an AAV injection involving the caudomedial portion (**A**) or the anterolateral portion (**E**) of GPi/ENT. Pallidothalamic axons labeled from the injection shown in panel A are limited to the most anterior part of VA-VL (**B**), the dorsal/medial portion of the parafascicular nucleus (Pf, panel **C**), and the medial portion of the lateral nucleus (LHbL, panel **D**). In contrast, the injection in the anterolateral portion of GPi/ENT did not labeled axons in the ventral thalamic nuclei (**F**) yet labeled a profuse plexus of axonal arborizations in the lateral/ventral portion of Pf (**G**) and the lateral part of the lateral habenular nucleus (LHbL, panel **H**). Images from the Mouse Connectivity Projection dataset https://connectivity.brain-map.org/projection/experiment/305024724 (**A**), /539498984 (**E**). Bregma level (in mm) is indicated in the upper right (**A-H**) corner. Scale bars: 200 µm (**A and E**), 250 µm (**B-D, F-H**).

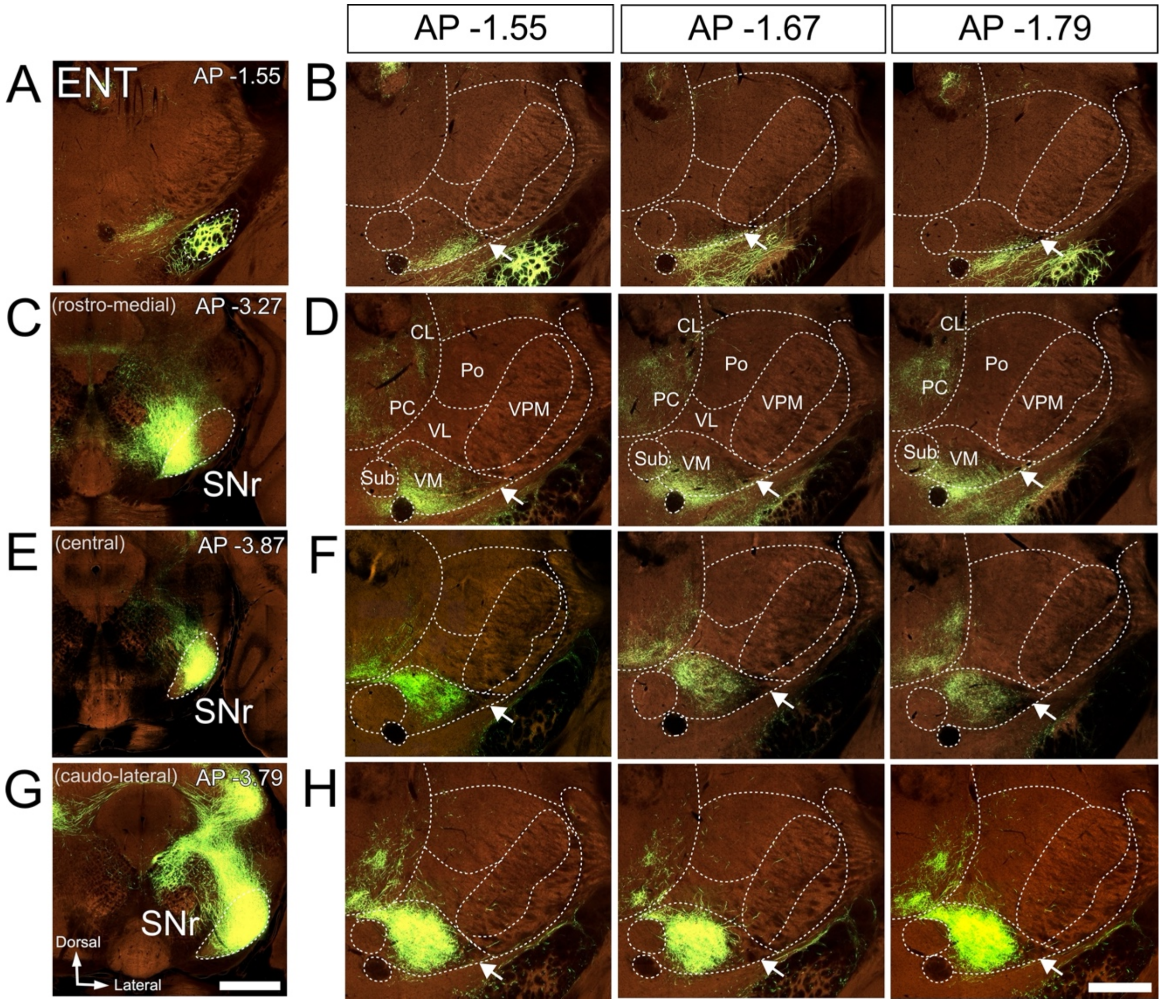

Supplementary Figure 3. Nigral and pallidal inputs avoid the caudolateral portion of the ventromedial nucleus.

Two-photon tomography image samples showing additional coronal section levels in the SNr and ENT experiments shown in Figure 7. Injection sites in ENT (**A-B**) and SNr (**C-H**). (**B, D, F and H**): Coronal section images showing axons labeled (green fluorescence). The white arrow points to the caudolateral portion of VM, which is mostly free of labeled fibers. Scale bars: 1mm (**A, C, E and G**), 500 µm (**B, D, F and H**).
